# Cortical Volume in the Right Cingulate Cortex Mediates the Increase of Self-Control From Young Adult to Middle-Aged

**DOI:** 10.3389/fnbeh.2022.723786

**Published:** 2022-04-01

**Authors:** Lili Jiang, Chunlin Li, Yubin Li

**Affiliations:** ^1^CAS Key Laboratory of Behavioral Science, Institute of Psychology, Beijing, China; ^2^Department of Psychology, University of Chinese Academy of Sciences, Beijing, China

**Keywords:** MRI, self-control, cortical volume, cingulate, aging

## Abstract

A high self-control capacity is related to better environmental adaptability and happy and healthy life. Neuroimaging studies have elucidated that the anterior cingulate, the prefrontal cortex, and the orbitofrontal cortex are involved in self-control. However, few studies integrated all three measurements, namely, age, human brain, and self-control, into a single quantitative model and examined whether self-control ability increased or decreased with age. In this study, we collected 65 participants’ data including structural MRI and Tangney’s Self-Control Scale to explore age dependence of cortical volume (CV) and self-control from young adult to middle-aged, as well as whether a non-linear association in the tridimensional model of age-brain-self-control was necessary to explain all the data in this study. We showed that self-control increased with age, but CV decreased with age. In a linear model, our mediation analyses revealed that CV in the right cingulate cortex mediated the increase of self-control; we also constructed a general non-linear model of age-brain-behavior and proved that the inverted development of human brain morphology and self-control abilities happened when morphology decays with age at a relatively smaller rate. Our study indicated that healthy aging in terms of increasing self-control is achievable, and our quantitative linear model of self-control laid theoretical foundations for studies on non-linear associations in age-brain-behavior.

## Introduction

Self-control is the ability to control one’s behaviors, actions, emotions, thoughts, and so on. Gaining good self-control over these aspects is a key developmental milestone across the life span. People with a high personal capacity of self-control should have better environmental adaptability and live happier and healthier lives. In more detail, they would do well in managing their lives, holding their tempers, keeping their diets, fulfilling their promises, stopping after a couple of drinks or smoking, persevering their work, and keeping their secrets (Tangney et al., 2004). The development of self-control is of course very important for children, teenagers, and adolescents (Casey and Caudle, 2013): self-control could forecast better psychosocial outcomes (Miller et al., 2015), health wealth, and public safety (Moffitt et al., 2011); disturbances in the maturation of self-control processes likely contribute to a variable of neuropsychiatric disorders (Marsh et al., 2007). Also, most studies on self-control focus on children and teenagers (Fjell et al., 2012; Casey and Caudle, 2013; Nelson et al., 2019). Consequently, considering (1) even from young adult to middle-aged, human brain and self-control capability still develop quite a lot, (2) multimodal and multi-method discrepancies in recent MRI studies of the human brain on the development of cortical morphology (Walhovd et al., 2016), a study using a relatively narrow age span, such as from young adult to middle-aged, is very necessary for further clarifying quantitative characteristics of the human brain and behavioral development.

How do human brain morphology and self-control ability develop from young adult to middle-aged? How does human brain morphology influence the development of self-control ability? There was still no study addressing this question in a quantitative way, and previous studies mostly employed self-control task-related fMRI to detect brain regions that were activated. Although Tang et al. (2015) have summarized a complete picture of the circuitry of self-control that included amygdala, dorsal anterior cingulate (dACC), dorsal prefrontal cortex (dPFC), *ventromedial prefrontal cortex* (vmPFC), and so on (Tang et al., 2015), no quantitative model of age-brain-behavior associations was proposed. Existing examinations on the neural mechanism of self-control were not a lot but included brain structure, brain function, and biochemical measurements. For brain structure, self-regulation was related to gray matter (GM) volume in the anterior cingulate cortex (Hashimoto et al., 2015), cortical surface in the anterior cingulate cortex (Fjell et al., 2012), structural frontostriatal connectivity strength (Hanggi et al., 2016), and maturation of the frontal and parietal cortices (Nelson et al., 2019). For brain function, self-control was associated with the prefrontal, the posterior cingulate cortices, and the frontostriatal regions in both healthy participants (Marsh et al., 2006) and patients with Tourette’s syndrome (Marsh et al., 2007), resting-state functional network dimension (Berman et al., 2013), negative functional coupling between the right frontoparietal and limbic resting-state networks (Lee and Telzer, 2016), and functional frontostriatal connectivity strength in healthy elderly (Hanggi et al., 2016). For biochemical measurement, the self-regulatory capability was related to the anterior cingulate, insula, and posterior cingulate using *N*-acetyl-L-aspartate (NAA) concentration (Nelson et al., 2019). Most of these studies only tested the associations of self-control with the human brain but did not combine self-control with both age and brain characteristics.

Aging was a rather complicated dynamic process. The development of both self-control and human brain was also complicated and dynamic. Although previous task-based self-control studies have already plotted developmental trajectories of self-control, they mostly used cognitive scores that focused on cognitive theory but rarely from the perspective of mental health. The Tangney’s Self-Control Scale (Tangney et al., 2004) focused more on mental health, and we could use it to measure self-control abilities in this study from a more general perspective because it was easier to collect by questionnaire. For human brain development, most studies recruited children and teenagers, and two review papers summarized the development of cortical thickness (Walhovd et al., 2016), surface area, and volume (Miller et al., 2015): decreasing from childhood to adolescence and then to young adult or a subtle increase in children and then a continuous decreasing from childhood to adolescence to young adult. Shaw et al. (2006) also depicted different trajectories of cortical thickness development for different intelligence groups, but they did not give any quantitative model of the three variables, age, human brain, and intelligence.

Recently, Schiavo et al. (2019) supposed a dynamic system approach to the triadic reciprocal determinism of social cognitive theory, including individual behavior, personal factors, and environmental challenges. Combining the abovementioned brain mechanisms of self-control and the dynamic modeling method, in this study, we proposed a tridimensional model of age-brain-self-control to study (1) the development of human brain morphology and self-control from young adult to middle-aged; (2) whether cortical volume (CV) worked as a mediator to affect the development of self-control; (3) when the inverted development of cortical morphology and self-control happened in a general model of age-brain-self-control. Focusing on such a special age range, 18–65 years old, our study would illustrate that healthy aging in terms of increasing self-control with age is achievable, and our quantitative linear model of behavior mediated by human brain morphology laid some theoretical foundations for studies on non-linear associations in age-brain-behavior (Jiang et al., 2018, 2019).

## Materials and Methods

### Participants

In total, 67 healthy subjects (32 men, aging from 18.6 to 64.3 years) were recruited from local community or universities *via* advertisements. All the participants were invited for a detailed mental health interview using the Mini-International Neuro-Psychiatric Interview. People with a history of major neuropsychiatric illness, head injury, alcohol, and drug abuse were excluded. They were also assessed with Wechsler Adult Intelligence Scale-4th Edition (WAIS-IV, in Chinese), Schutte Self-Report Emotional Intelligence Scale (SSEIS, in Chinese version), and Tangney’s Self-Control Scale (Tangney et al., 2004). Tangney’s Self-Control Scale comprises of five subscales, namely, impulse control (IC), healthy habit, reject temptation, job focus, and control entertainment. The institutional review board of the Institute of Psychology Chinese Academy of Sciences approved this study, and written informed consent was obtained from individuals prior to data acquisition.

### MR Images

All the MR images were collected on the 3.0 T GE Discovery MR750 at the Institute of Psychology Chinese Academy of Sciences. All the participants completed a T1-weighted structural MRI (sMRI) scan (eyes closed) with a ABI1_t1iso_fspgr sequence [repetition time (TR) = 6.652 ms; echo time (TE) = 2.928 ms; flip angle (FA) = 12°; matrix = 256 × 256; slice thickness = 1 mm; 192 sagittal slices; field of view = 256 × 256 mm^2^, and voxel size = 1 × 1 × 1 mm^3^].

### Image Preprocessing

All the images were preprocessed by the Connectome Computation System (CCS), which was integrated by our laboratory using FSL, AFNI, and FreeSurfer (Xu et al., 2015). Its significant characteristic is that it focuses on surface-based analysis compared with other resting-state fMRI data analysis software. Detailed information on the system could be found in our previous publications (Jiang et al., 2015). As we have analyzed human brain morphology, only structural image preprocessing was used in this study. The structural image preprocessing was conducted mainly by cortical surface reconstruction (Dale et al., 1999; Fischl et al., 1999), which included (1) T1 image noise removal and brain extraction by volBrain automated volumetry system^1^ (Manjon and Coupe, 2016), (2) segmentation of cerebrospinal fluid (CSF), white matter (WM), and GM, (3) construction of the GM-WM interface (white surface) and the GM-CSF interface (pial surface), and (4) spatial registration *via* matching of the cortical folding patterns across subjects. Finally, individual preprocessed sMRI data were projected onto the *fsaverage5* standard cortical surface with 10,242 vertices per hemisphere and an average spacing of about 4 mm (Yeo et al., 2011).

### Quality Control

Quality control was the most important step during data analysis. In this study, we considered brain extraction, pial, and white surface reconstruction for quality control. We acquired screenshots and check their qualities by visually checking. One participant did not finish MRI scanning, and one participant did not pass the mental health interview. Thus, we have 65 participants for the final group analysis. The detailed participant information and behavioral measurements are summarized in Table 1. Participants with the higher scores of each subscale have better self-control abilities.

**TABLE 1:** Participant information.

Measurements	Average ± SD	Range
Age (years)	37.3 ± 13.1	18.6–64.3
Sex (male/female)	32/33	N/A
Education (years)	15.4 ± 3.2	8–22
Impulse control	23.0 ± 4.7	13–30
Healthy habit	10.3 ± 2.8	5–15
Reject temptation	13.3 ± 2.8	5–20
Job focus	10.1 ± 2.5	5–15
Control entertainment	10.6 ± 2.9	4–15

### Group Analysis

Considering recent discussions on multiple comparison correction of voxel-wise fMRI studies (Eklund et al., 2016; Brown and Behrmann, 2017; Cox et al., 2017; Kessler et al., 2017), in this study, we used a large-scale Yeo 7 network (Yeo et al., 2011) parcelation (51 parcels) to examine the development of human brain morphology (CV), as well as the associations of brain morphology and self-control abilities. We believed that the large-scale parcelation might help to reduce fMRI noise and artifacts and increase the statistical power and reliability of our results. We used the MATLAB corr function to calculate the effects of age on human brain CV and self-control. According to the mediation model described by Wen and Ye (Wen and Ye, 2014), we first testified the significance of age effects on self-control (c) and CV (a), as represented in Equations 1, 2). Then, we used partial correlation to test the associations of CV with self-control (b) (as represented in Equation 3), excluding the effects of age, sex, education, and estimated Total Intracranial Cortical Volume (eTICV). Finally, we used the Bonferroni method for multiple comparison correction of multiple brain regions. We also calculated the replication rate of the association study using the leave-one-subject-out algorithm.


(1) $$S\,e\,l\,f\,c\,o\,n\,t\,r\,o\,l=c*A\,g\,e+e_{1}$$

(2) $$V\,o\,l=a*A\,g\,e+e_{2}$$

(3) $$S\,e\,l\,f\,c\,o\,n\,t\,r\,o\,l=c^{\prime }*A\,g\,e+b*V\,o\,l+\beta_{1}*S\,e\,x+\beta_{2}*e\,d\,u+\beta_{3}*e\,T\,I\,C\,V+e_{3}$$

## Results

### Five Dimensions of Self-Control All Increased With Age

**FIGURE 1:**
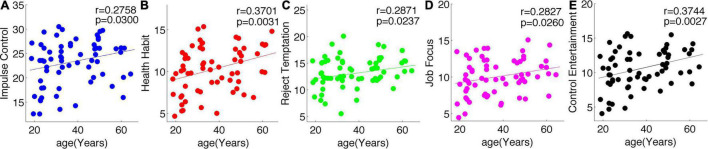
All the five subscales of self-control increased with age. Blue, Impulse Control **(A)**; red, Health Habit **(B)**; green, Reject Temptation **(C)**; purple, Job Focus **(D)**; black, Control Entertainment **(E)**.

As shown in Figure 1, all the five dimensions of self-control, namely, IC, healthy habit, reject temptation, job focus, and control entertainment, increased with age.

### Decreased Cortical Volume in the Frontoparietal Control Network and Default Network With Age

**FIGURE 2:**
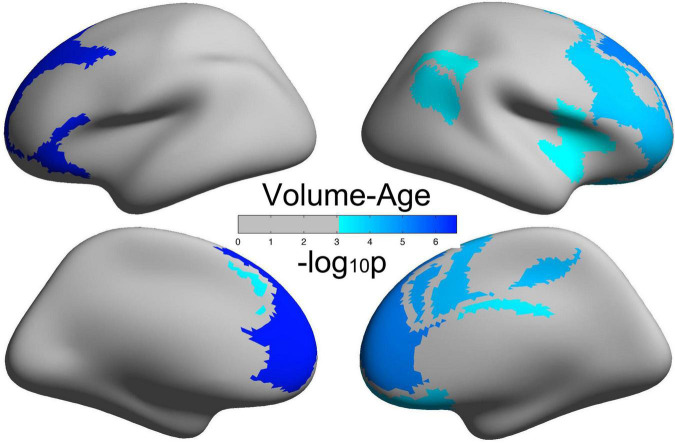
Brain regions with a significantly decreased cortical volume (CV) of the human brain with age.

Figure 2 illustrates brain regions with significant negative correlations of CV with age. Detailed information of these brain regions is summarized in Table 2, mainly including the frontoparietal control network and the default network.

**TABLE 2:** Brain regions with significant negative correlations between cortical volume and age (*p* < 0.05/51).

Brain regions	Abbreviations	Number of vertex	*r*	*p*-value
Left_Control_PreFrontalCortex_medialposterior	L_Cont_PFCmp	21	−0.4192	5.0871e-04
Left_Default_ PreFrontalCortex	L_Default_PFC	771	−0.5899	2.3309e-07
Right_SalientVentralAttention_FrontalOpercular	R_SalVentAttn_FrOper	313	−0.4036	8.5631e-04
Right_SalientVentralAttention_Medial	R_SalVentAttn_Med	242	−0.4779	5.6823e-05
Right_Limbic_OrbitalFrontalCortex	R_Limbic_OFC	237	−0.4496	1.7196e-04
Right_Control_Parietal	R_Cont_Par	167	−0.4058	7.9635e-04
Right_Control_PreFrontalCortexlateral	R_Cont_PFCl	543	−0.4710	7.5166e-05
Right_Control_Cingulate	R_Cont_Cing	47	−0.4246	4.2320e-04
Right_Control_PreFrontalCortexmedialposterior	R_Cont_PFCmp	48	−0.4969	2.5512e-05
Right_Default_Parietal	R_Default_Par	183	−0.4256	4.0920e-04
Right_Default_PreFrontalCortexmedial	R_Default_PFCm	461	−0.5265	6.6558e-06

### Cortical Volume Worked as a Mediator to Link Age and Self-Control

**FIGURE 3:**
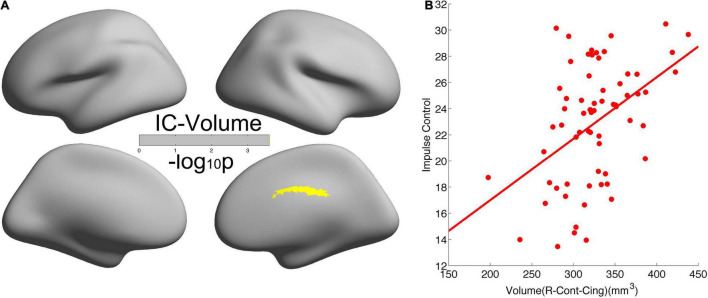
Significant positive correlation of cortical volume in the right cingulate with self-control. **(A)** Brain regions with significant correlations between cortical volume and self-control. **(B)** Scatter of the positive correlation.

**FIGURE 4:**
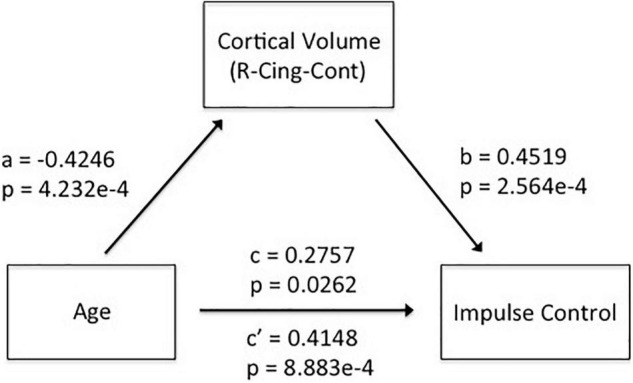
Mediation analyses revealed that CV in the right cingulate mediated the adult age differences in self-control.

As shown in Figure 3, we found significant positive correlations of CV in the right cingulate with IC (*r* = 0.4519, *p* = 2.5638e-4). The leave-one-subject-out algorithm demonstrated that this partial correlation exhibited a replication rate of 98.46%. Integrating the significant age effects on self-control and CV, the mediation analyses revealed that CV mediated the age dependence of self-control from young adults to middle-aged, as summarized in Figure 4.

### A Non-linear Model of Age-Brain-Behavior

Although the abovementioned results demonstrated that a linear model could account for the inverted developments of human brain morphology and self-control, in this study, we still constructed a general non-linear model of behavior for future theoretical reference. Since, at present, we only detected IC as a unique variable varying with age and CV, we constructed a model of IC taking the lower level measurement “human brain morphology (CV)” as a median variable and the lowest level measurement “age” as an independent variable as follows:


(4) $$I\,C=f_{1}\,\left(a\,g\,e\right)+f_{2}\,\left(C\,V\right)+f_{3}\,\left(a\,g\,e\right)\,f_{4}\,\left(C\,V\right)+\beta$$

(5) C⁢V=f⁢(a⁢g⁢e)

The slope of IC changing with age should be as follows:


(6) $$\frac{d\,I\,C}{d\,a\,g\,e}=\frac{\partial f_{1}}{\partial a\,g\,e}+\frac{\partial f_{2}}{\partial C\,V}\,\frac{\partial C\,V}{\partial a\,g\,e}+f_{4}\,\frac{\partial f_{3}}{\partial a\,g\,e}+f_{3}\,\frac{\partial f_{4}}{\partial C\,V}\,\frac{\partial C\,V}{\partial a\,g\,e}=\frac{\partial I\,C}{\partial a\,g\,e}+\frac{\partial I\,C}{\partial C\,V}\,\frac{\partial C\,V}{\partial a\,g\,e}$$

When we used linear correlation to study the associations of IC with age, IC with CV, or CV with age, we just, respectively, got partial correlation $\frac{\partial I\,C}{\partial a\,g\,e}$, $\frac{\partial I\,C}{\partial C\,V}$, or $\frac{\partial C\,V}{\partial a\,g\,e}.$ Considering our results in this study, $\frac{\partial I\,C}{\partial a\,g\,e}>0$, $\frac{\partial I\,C}{\partial C\,V}>0$, $\frac{\partial C\,V}{\partial a\,g\,e}<0$, as well as Equation 6 should also be bigger than zero, so the following equation is needed:


(7) $$\left|\frac{\partial C\,V}{\partial a\,g\,e}\right|<\frac{\partial I\,C}{\partial a\,g\,e}/\frac{\partial I\,C}{\partial C\,V}$$

So far, if Equation 7 was satisfied, then the inverted developments of brain morphology and self-control would be consistent with the positive correlations of brain morphology with self-control abilities. Equation 7 meant that human brain morphology CV decays with age at a smaller rate than the ratio of the slope of IC with age and the slope of IC with CV. In this study, the significant association of IC with CV satisfied Equation 7: $\left|\frac{\partial C\,V}{\partial a\,g\,e}\right|=0.2928<0.4148/0.4519,$ wherein $\frac{\partial I\,C}{\partial a\,g\,e}=0.4148,\frac{\partial I\,C}{\partial C\,V}=0.4519$.

Although we did not depict a more detailed age-brain-behavior model, we gave a quantitative conclusion on the non-linear associations among age, brain morphology, and self-control, which was that the inverted development of human brain morphology and self-control abilities happened when morphology decays with age at a relatively smaller rate. Considering most of the studies used linear statistics, such kind of non-linear model would definitely promote the applications of non-linear perspective in future human brain studies (Jiang et al., 2018, 2019).

## Discussion

In this study, we demonstrated that self-control and human brain CV inversely developed from young adult to middle-aged: all the five dimensions of self-control, namely, IC, healthy habits, reject temptation, job focus, and control entertainment, increased with age, but CV decreased with age in the frontoparietal control network and default network. Meanwhile, we observed significant positive correlations of CV with IC in the right cingulate. Such inverted changes in brain morphology and behavior as well as the positive brain-behavior correlations promoted us to propose a mediation model of the tridimensional age-brain-behavior dynamic system. Although a non-linear model was not necessary for the associations of age-CV-IC in this study, our linear model and the general non-linear model shed new lights on future non-linear studies on the human brain.

### Inverted Changes of Brain Morphology and Self-Control From Young Adult to Middle-Aged

Middle-aged and young adults are both adults and are generally taken as one single group in brain-behavior association studies in comparison with the elderly group. In this study, we aimed to examine the early aging effects of self-control and brain morphology from young adults to middle-aged. First, we have demonstrated that all the five dimensions of self-control increased with age; such healthy aging in the middle-aged was first proposed in the perspective of mental health and may shed new lights on mental health studies in the middle-aged but not only in the elderly. Second, our results showed that CV in the frontoparietal control network and the default network decreased with age, and the prefrontal cortex showed the largest aging effects. Such decreasing trend of human brain morphology with age was consistent with previous studies (Mills and Tamnes, 2014; Walhovd et al., 2016), and its specific mechanisms were still not understood well, possibly due to the proliferation of myelin and elimination of synapses (Sowell et al., 2004; Petanjek et al., 2011). Third, the right cingulate cortex, as a part of the control network, exhibited decreased CV from young adults to middle-aged. Also, the cingulate cortex had been testified to play key roles in self-control abilities (Marsh et al., 2006, 2007). Compared with the most striking reductions of CV with age in the prefrontal cortex, the cingulate cortex exhibited less aging effects on the decreasing of CV. This finding was not only consistent with other studies (Grieve et al., 2005; Lemaitre et al., 2012) but also verified the “last in, first out” hypothesis that the brain regions that were finally matured or developed were the first to be affected by aging. All the above mentioned studies examined the associations of only two variables: age vs. human brain, age vs. self-control, or human brain vs. behavior. Consequently, an integrative model of age-brain-behavior may better elucidate the mechanisms rooted in the development of human brain morphology and self-control.

### Roles of the Right Cingulate Cortex in Self-Control

In this study, we found a significant positive correlation of CV in the right cingulate with IC, and this correlation had a very good leave-one-subject-out replication rate. These validated that the cingulate cortex played key roles in self-control, and also, this conclusion was rather reliable. The anterior cingulate cortex was reported in most studies on self-control (Marsh et al., 2006; Tarullo et al., 2009; Fjell et al., 2012; Tang et al., 2015). Actually, the parcelation we reported here included some vertices in the anterior and posterior cingulate cortex, and most vertices were in the middle cingulate cortex. Since the realistic exact neural underpins of self-control were still on debate, at present, we believed here that the three subregions of the cingulate cortex all took some parts in self-control. First, the anterior cingulate cortex received messages from many brain regions and integrated all of the information to regulate both cognitive and emotional processes (Zelazo et al., 2008). Similarly, the surface area of the anterior cingulate cortex accounted for a significant proportion of the variance in cognitive performance (Fjell et al., 2012). Second, the posterior cingulate cortex was proposed to support internally directed cognition (Leech and Sharp, 2014), as well as regulated spatial and action-related information from parietal cortical areas to hippocampal systems (Rolls, 2019). Self-control was just an internally directed decision, and before making a “wise” self-control-related decision, one had to retrieve spatial, action, and memory-related information and had to make cognitive and social inferences (Buckner et al., 2008; Buckner and DiNicola, 2019). Third, the midcingulate cortex resides between the anterior cingulate and the posterior cingulate. By assisting both the anterior cingulate and the posterior cingulate, the midcingulate cortex enabled reward outcome information from the orbitofrontal cortex (OFC) to be associated with action information from the posterior cingulate cortex (Rolls, 2019).

In this study, we observed that only the right cingulate cortex exhibited significant mediation effects of CV on the associations between age and self-control but not in the left hemisphere. There were also two other studies reporting the right cingulate cortex survived multiple comparison corrections in self-control studies (Casey et al., 1997; Fjell et al., 2012). However, we need more studies to validate the existence of hemispheric asymmetry.

### Cortical Volume in the Cingulate Cortex as a Target Variable in Future Models of Age-Brain-Behavior Studies

Elucidating the associations of age-brain-behavior is the ultimate aim of neuroscience research. Besides several studies addressing neural mechanisms of self-control (Marsh et al., 2006; Fjell et al., 2012; Berman et al., 2013; Hashimoto et al., 2015; Lee and Telzer, 2016), our study demonstrated that CV in the cingulate cortex worked as an intermediating variable to affect the associations of self-control with age. This mediation model supplied us with a linear perspective to understand the associations of age-brain-behaviors. Although this linear association is the simplest description of the tridimensional dynamic system, it could deepen our understanding of the complicated system, and gave us some pioneer ideas to construct a more complicated model of age-brain-behaviors. Furthermore, we constructed a general non-linear model of age-CV-IC, and we gave a quantitative conclusion, which was that the inverted development of human brain morphology and self-control abilities happened when morphology decays with age at a relatively smaller rate. Considering most of the studies used linear statistics, such kind of non-linear model would definitely promote the applications of non-linear perspective in future human brain studies.

### Limitations

There were some limitations in this study. First, we used cross-sectional data to study the development of human brain and self-control abilities. Longitudinal data would be better to characterize the intra- and intersubject variations in human brain and self-control as well as the causality reasoning among age, human brain, and self-control (Wen, 2017). Second, our sample size was not big, and more participants, especially children and adolescents, may help us find some more precise developmental trajectory of both human brain morphology and self-control.

## Conclusion

We observed that self-control and human brain morphology inversely developed from young adult to middle-aged: all the five subscales of self-control increased with age, but CV decreased with age. In a linear model, our mediation analyses revealed that CV in the right cingulate mediated the increase of self-control from young adult to middle-aged; we also constructed a general non-linear model of age-brain-behavior and proved that the inverted development of human brain morphology and self-control abilities happened when morphology decays with age at a relatively smaller rate. The increased self-control with age indicated that healthy aging is achievable, and our quantitative linear model of behavior mediated by human brain morphology laid some theoretical foundations for studies on non-linear associations in age-brain-behavior.

## Data Availability Statement

The raw data supporting the conclusions of this article will be made available upon request from the corresponding author.

## Ethics Statement

The studies involving human participants were reviewed and approved by the institutional review board of Institute of Psychology, Chinese Academy of Sciences. The patients/participants provided their written informed consent to participate in this study.

## Author Contributions

LJ: conceptualization and methodology. LJ and CL: formal analysis and investigation. LJ, CL, and YL: writing-review and editing. All authors contributed to the article and approved the submitted version.

## Conflict of Interest

The authors declare that the research was conducted in the absence of any commercial or financial relationships that could be construed as a potential conflict of interest.

## Publisher’s Note

All claims expressed in this article are solely those of the authors and do not necessarily represent those of their affiliated organizations, or those of the publisher, the editors and the reviewers. Any product that may be evaluated in this article, or claim that may be made by its manufacturer, is not guaranteed or endorsed by the publisher.

## Footnotes

1
https://www.volbrain.upv.es
